# Supplementary material for: Multivoxel neurofeedback selectively modulates confidence without changing perceptual performance
Source: Nat Commun. 2016 Dec 15;7:13669. doi: 10.1038/ncomms13669 (PMC5171844; doi:10.1038/ncomms13669)
Supplement: Supplementary Information — Supplementary Figures 1-11, Supplementary Tables 1-7, Supplementary Note 1 and Supplementary References [file ncomms13669-s1.pdf]

# Supplementary Figures

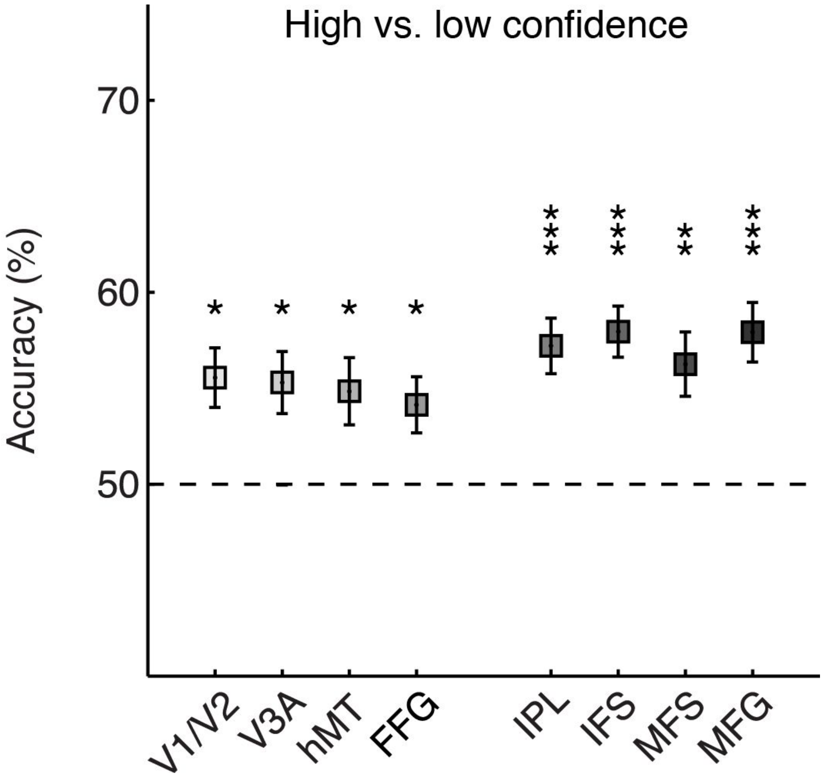


**Supplementary Figure 1. Control classification of High- vs. Low-Confidence**

The control analysis uses all samples, with $n$ = 10 random sampling sets, and for each participant the final accuracy was computed by averaging the mean cross-validated accuracy of each sampling set. The same pattern of results was found; namely, decoding of perceptual confidence was higher in frontoparietal areas as compared to visual processing regions.

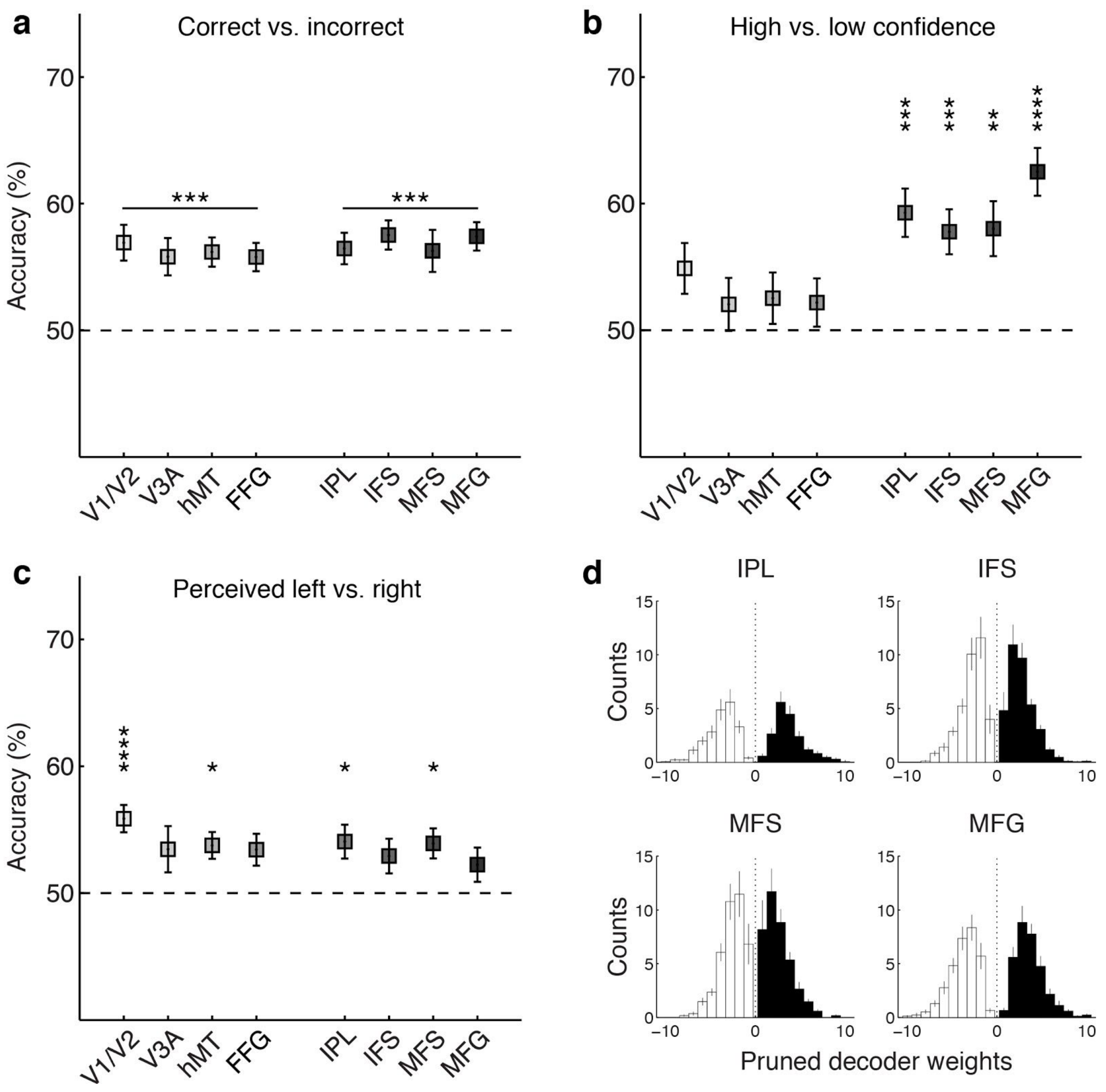


**Supplementary Figure 2. MVPA results controlled for ROI sizes**

(a-d) For each subject, the ROI with the lowest number of voxels was first defined. Then, for all other ROIs, this voxel number was selected based on the highest absolute *t*-values following an univariate analysis, with contrast stimulus vs. blank baseline. Accuracy in classifying (a) correct vs. incorrect trials, (b) high- vs. low-confidence trials, and (c) perceived motion direction (left vs. right). (d) Pruned weights of the confidence decoder. Qualitatively, the same pattern of results as in the main analysis was found. $n$ = 17, *$P$<0.05, **$P$<0.01, ***$P$<0.005, ****$P$<$10^{-3}$, *****$P$<$10^{-4}$; $P$ values corrected for multiple comparisons (Holm-Bonferroni). Center values correspond to means, and error bars to s.e.m. ROIs: V1/V2, V3A, hMT, FFG - fusiform gyrus, IPL - inferior parietal lobule, IFS - inferior frontal sulcus, MFS - middle frontal sulcus, MFG - middle frontal gyrus.

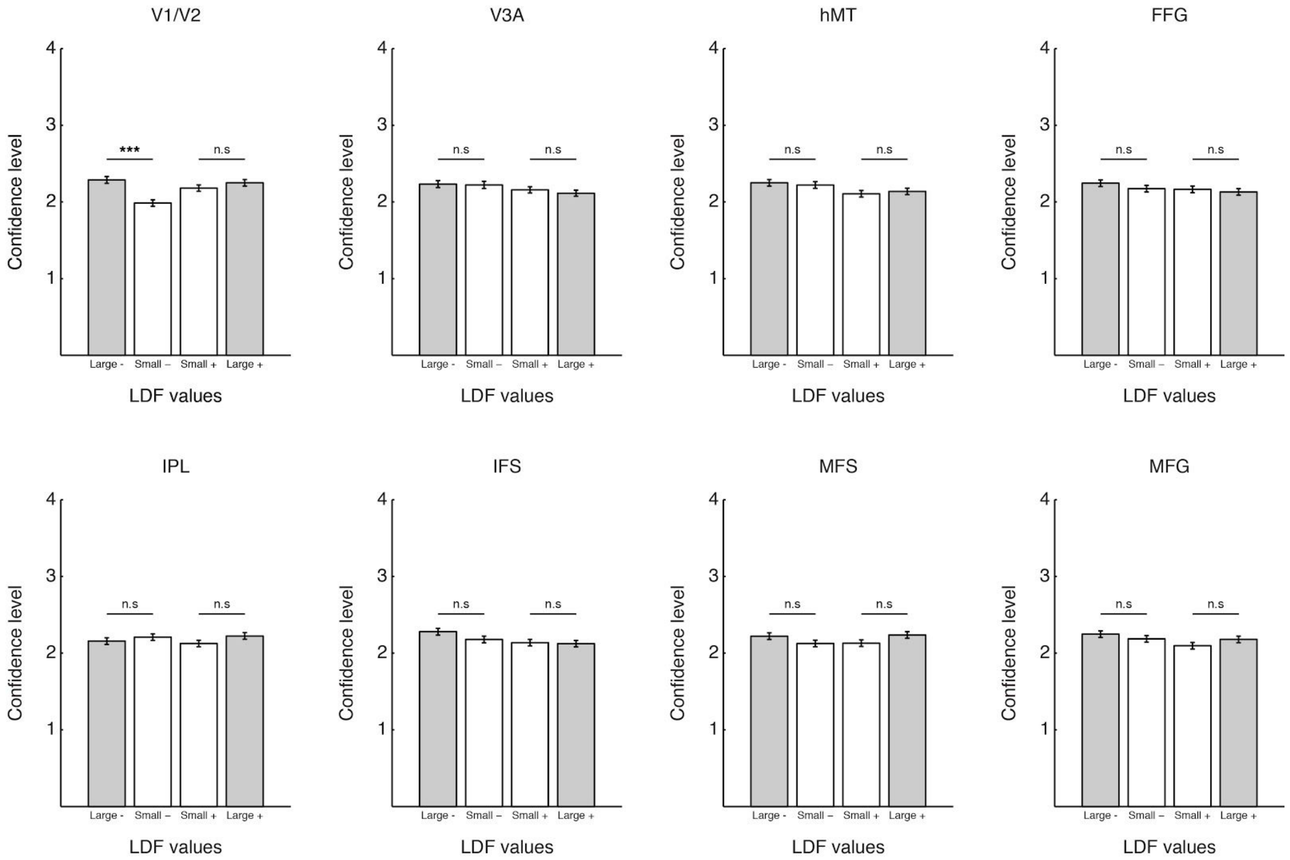


**Supplementary Figure 3. Relationship between linear classifier output (leftward vs. rightward motion) and confidence**

Here, negative and positive values of the classifier output (LDF values) corresponds to leftward and rightward motion, respectively. Reflecting the results in Fig. 6, there was no difference in confidence level as a function of the absolute classifier output (large or small) in most of the ROIs in either direction (left or right). Only exception was V1/V2 where the magnitude of the leftward classifier output (negative LDF) was related with a significant difference in confidence. For all other ROIs, the confidence levels did not significantly differ between the two levels of LDF magnitude (large or small). The threshold to define large vs. small LDF was defined as the median at the group level for each ROI. Center values represent mean, error bars represent s.e.m. ***$P<10^{-3}$, paired t-test between large/small LDF magnitude, corrected for multiple comparisons (two, negative/positive LDF values).

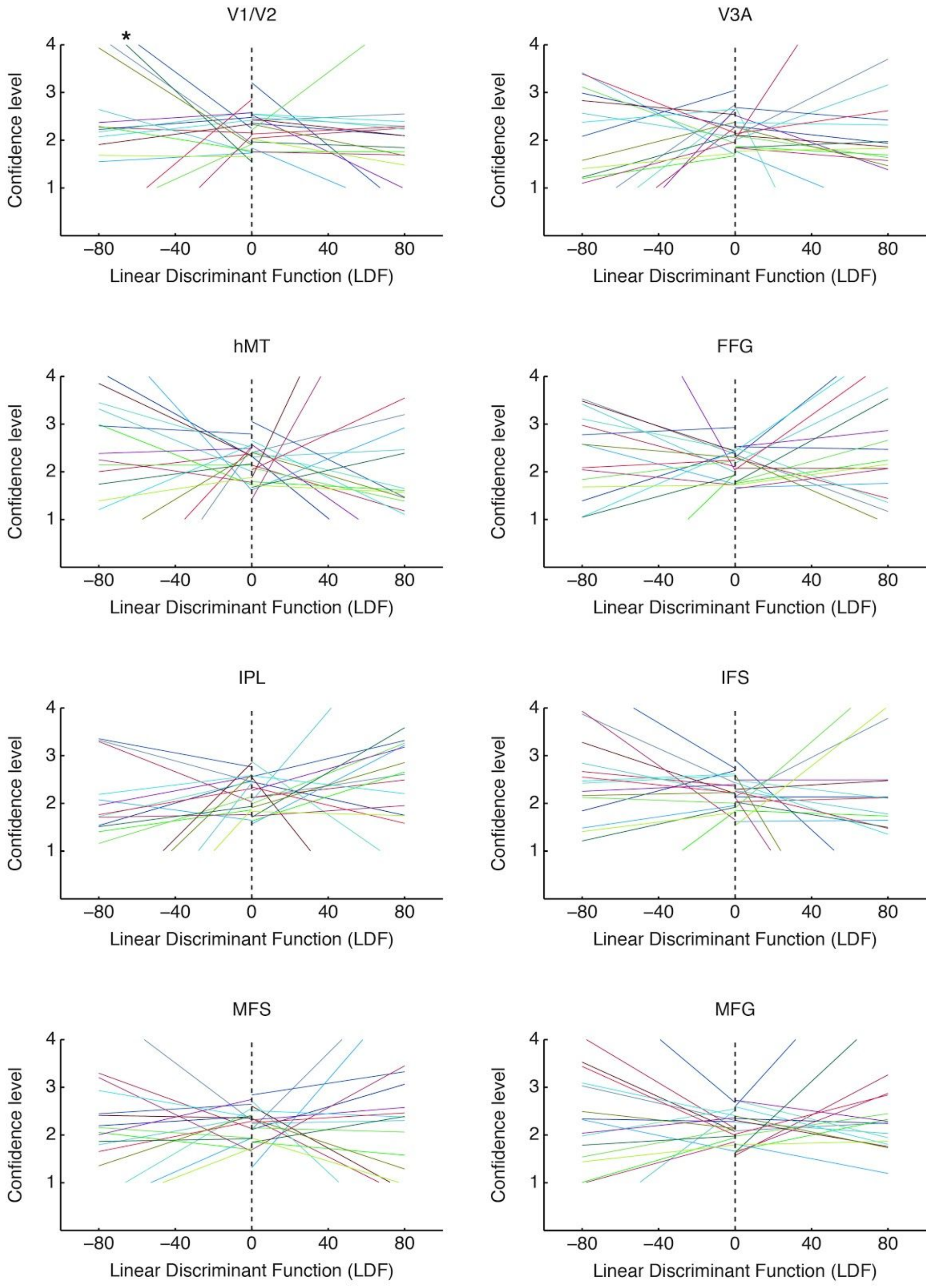


**Supplementary Figure 4. Subject level analysis of the relationship between linear classifier output (leftward vs. rightward motion) and confidence**

In accordance with the results at the group level (Fig. 6), there was no significant correlation between negative LDF and confidence levels in most of the ROIs, with only one exception in V1/V2 (corrected for multiple comparisons across participants). For each participant two linear fits were plotted, one for leftward motion (negative LDF) and the other for rightward motion (positive LDF).

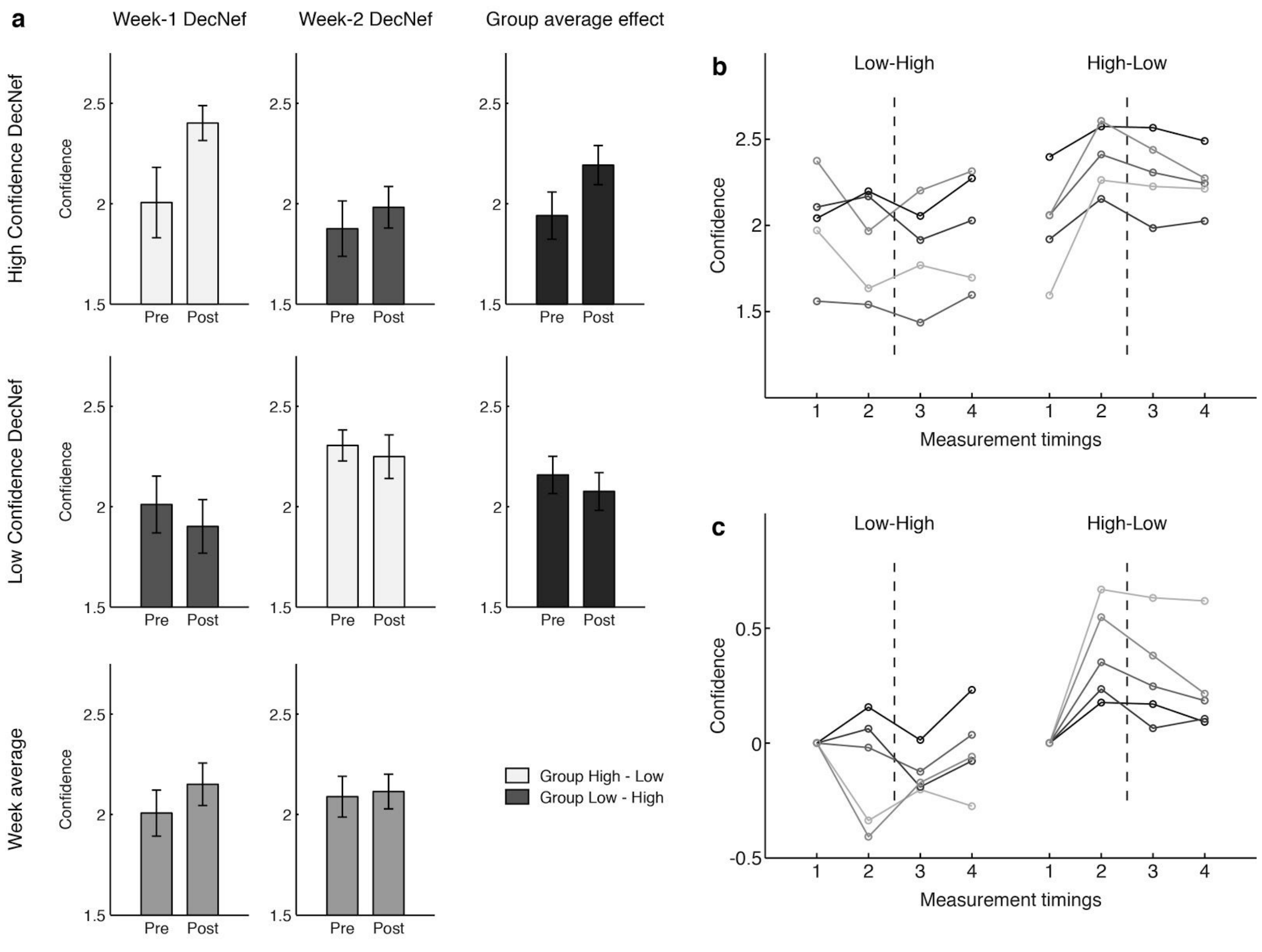


**Supplementary Figure 5. Confidence ratings in psychophysical Pre- and Post-Tests**

(a) Each group induced both High then Low Confidence, in two separate sessions: dark-gray colored group with the order Low then High Confidence, and light-gray colored group High then Low Confidence. Average across groups, representing mere order effect, is depicted at the bottom, while the grand average per condition (High and Low Confidence), is in black, on the right side. It is apparent that the confidence changes in the first week influenced the confidence level in the second week. The Pre- level in the second week remained very close to the previous Post-level attained in the first week. Furthermore, neurofeedback in the first week seemed to have a stronger impact than in the second week, and this holds when comparing both High and Low Confidence DecNef. For each bar, $n$ = 5. Error bars represent s.e.m. (b-c) participants' individual confidence data with the four measurement timings, organized by order groups (Low - High and High - Low), with both raw (b) and 0-aligned (c) confidence. Timings 1 and 2 correspond to Pre and PostTest in the first week, respectively; timings 3 and 4 to Pre and PostTest in the second week DecNef. The dotted line represents the weeklong interval between the two sessions (DecNef in week 1 and DecNef in week 2). Each small circle represents day-averaged data from one subject.

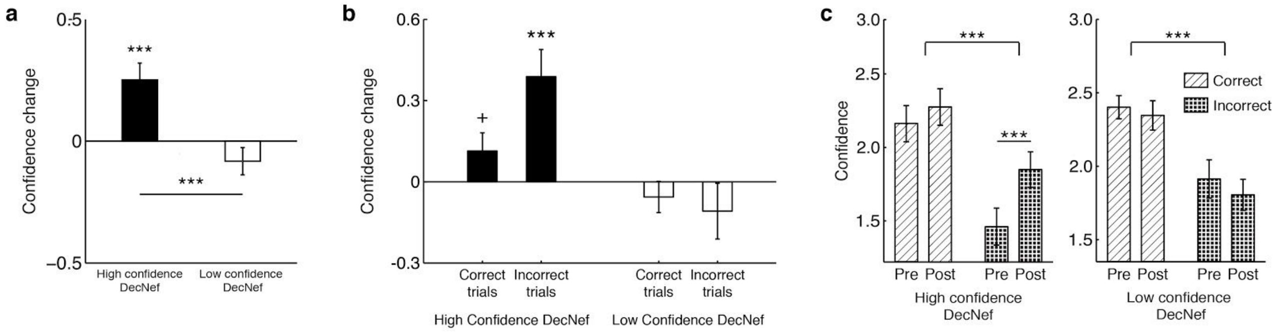


**Supplementary Figure 6. Bi-directional raw confidence changes**

(a) Bi-directional raw changes in High- and Low Confidence DecNef. The change was significant in the up-direction (High Confidence DecNef, one-sided t-test for increase, $t_9$ = 3.633, $P$ = 0.0027), and close to significance in the down-direction (Low Confidence DecNef, one-sided t-test for decrease, $t_9$ = -1.478, $P$ = 0.087). The difference High- vs. Low Confidence DecNef was also significant (one-sided t-test, $t_9$ = 3.672, $P$ = 0.0026). (b) Raw confidence changes for correct and incorrect trials, in High- and Low Confidence DecNef. Confidence change for incorrect trials in High Confidence DecNef was significantly different from 0 (one-sided t-test, $t_9$ = 3.879, $P$ = 0.0019), while in correct trials the change was close to significance (one-sided t-test, $t_9$ = 1.699, $P$ = 0.062). For Low Confidence DecNef the changes were not statistically significant (one-sided t-test, correct trials $t_9$ = -0.977, $P$ = 0.177; incorrect trials $t_9$ = -1.052, $P$ = 0.16). Nevertheless, a clear trend emerges from these data, indicating that the effect had opposite effects in High- and Low Confidence DecNef, and that it was larger for incorrect trials. (c) Confidence changes presented for High and Low Confidence DecNef, separated in “correct trials” and incorrect trials” categories. Raw values are reported, measured in the Pre- and Post- psychophysical tests. A three-way repeated measures ANOVA (factors of response accuracy [correct vs. incorrect], neurofeedback, and time) resulted in a significant three-way interaction ($F_{1,9}$ = 9.935, $P$ = 0.012), explicitly showing that confidence changed asymmetrically for correct and incorrect responses. Furthermore, there was also a significant main effect of response accuracy ($F_{1,9}$ = 28.942, $P$ = 0.0004). Post-hoc t-tests to contrast the Pre- versus Post-Tests showed a larger effect of DecNef on confidence ratings on incorrect trials (High Confidence DecNef, correct trials, $t_9$ = 1.7, $P$ = 0.124, incorrect trials, $t_9$ = 3.88, $P$ = 0.0037; Low Confidence DecNef, correct trials $t_9$ = -0.98, $P$ = 0.354, incorrect trials, $t_9$ = -1.05, $P$ = 0.32).

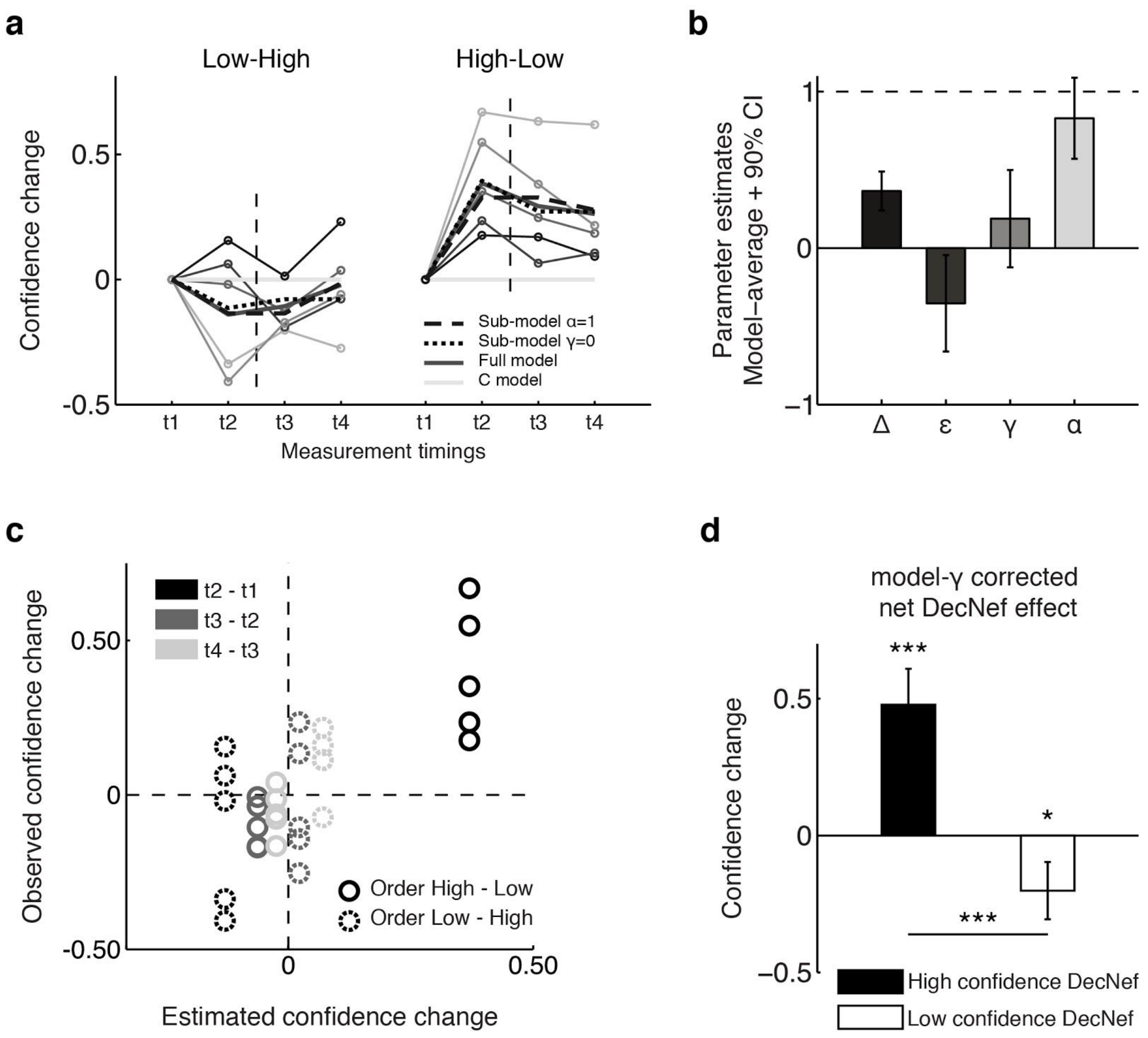


**Supplementary Figure 7. Nonlinear mathematical modeling**

Nonlinear mathematical modeling formally indicates the bi-directional nature of DecNef effects on confidence. (a) Individual and group-modeled data, 0-aligned. Thick achromatic lines represent different model fits, darker lines being better fits. The light gray line is the simplest model, assuming constant confidence (C model, 1 parameter model). The three other fits are the full model and two other models for which the computed AICc was the most negative or having a $\Delta_{AICc}$ < 2 from the most negative. Actual data from each participant is shown as a thin achromatic line with circles. (b) Global parameters estimates resulting from model averaging of the three best models. For each model, based on the $\Delta_{AICc}$, Akaike weights were computed. Each parameter was then evaluated as the weighted average of single model estimates. It is important to note that ε was both of opposed sign as compared with Δ, and different than zero. These two conditions give proof to the fact that DecNef induced confidence changes were bi-directional. Error bars represent 90% confidence intervals, computed from the unconditional standard error. (c) Plot of observed

confidence changes vs. model-based estimated confidence changes. There are 30 time points (change between t2 and t1, t3 and t2, t4 and t3), 3 for each of the 10 participants, divided between order groups (High - Low Confidence DecNef, and Low - High Confidence DecNef). The correlation between the two measures was highly significant (Pearson's $r$ = 0.748, $P < 10^{-5}$), supporting the validity of the estimated parameters. (d) Bi-directional net effects of High- and Low Confidence DecNef. The analysis takes into account the order and interference of DecNef sessions and integrates the estimated gamma parameter. The changes were significant for both High and Low Confidence DecNef directions, mathematically demonstrating that confidence successfully increased and decreased after neurofeedback training. $n$ = 10, *$P$<0.05, ***$P$<0.005, one-sided t-tests.

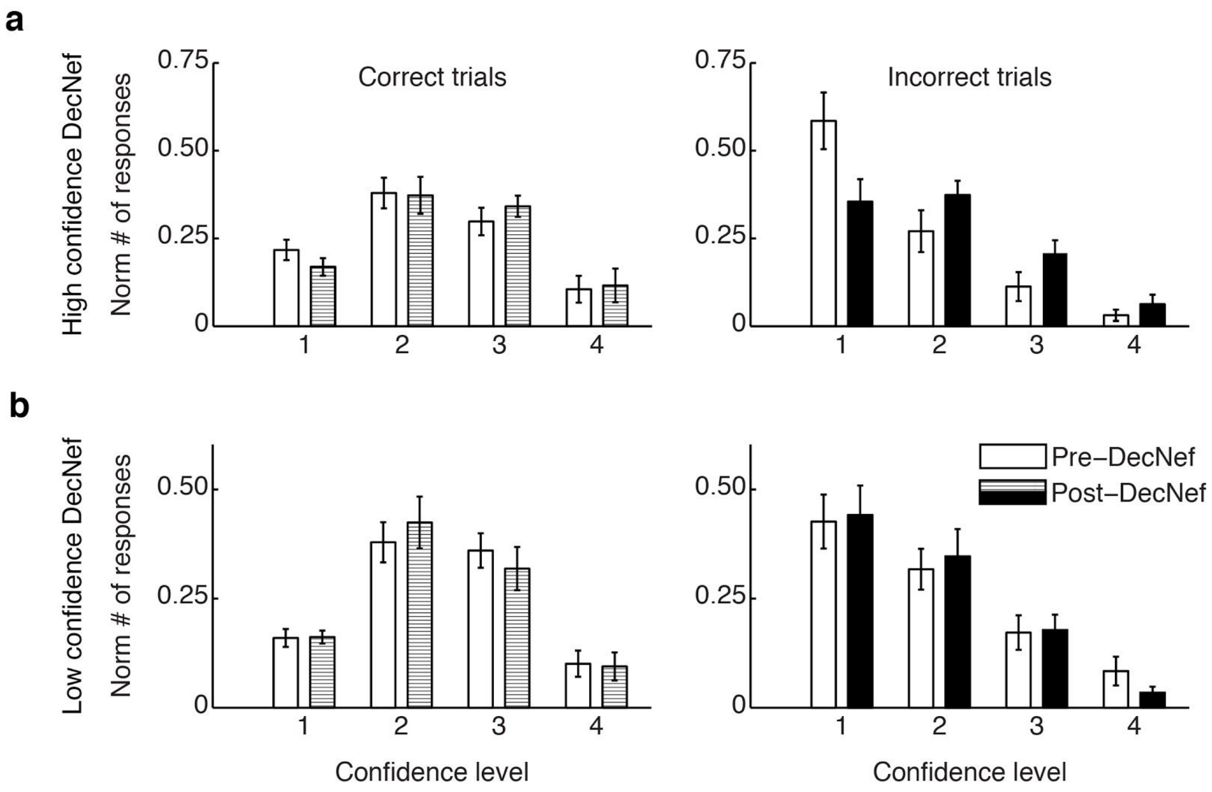


**Supplementary Figure 8. Detailed effects of DecNef on correct and incorrect trials**

Normalized response counts for each confidence level in the correct and incorrect categories, for High Confidence DecNef (a) and Low Confidence DecNef (b). Note the difference in choices in Pre- and Post-Tests: changes in confidence ratings were not symmetrical between levels. For each bar, *n* = 10. Center values correspond to means, and error bars to s.e.m.

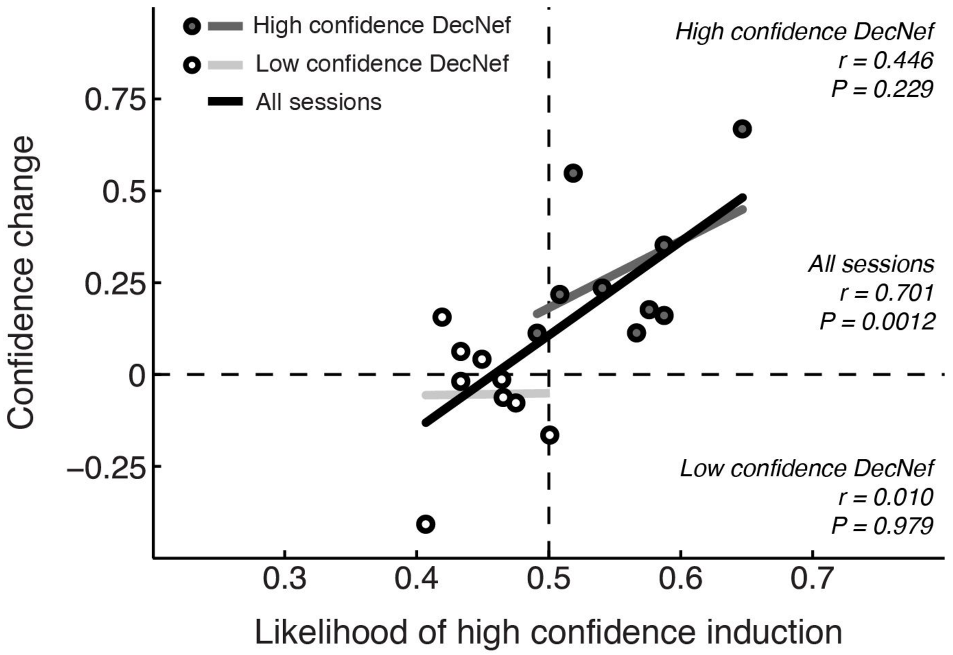


**Supplementary Figure 9. Correlation between neurofeedback induction success and confidence change**

Confidence change between Pre- and Post-Tests correlated with induction success on combined DecNef on day 1. The ordinate represents the change in confidence between Pre- and Post-Tests. The abscissa, the likelihood of high confidence induction, indexes neurofeedback success, 0.5 being the null point, with no effect expected. Values below 0.5 translate into higher likelihood of low confidence induction. Each data point represents one subject - since all participants performed in two DecNef blocks, there are 20 data points - averaged across trials and runs performed on day 1 of each session. Correlations were inferred by computing Pearson's *r*. Outliers, one in each group, were removed according to the criterion: $o \leq m - 2 * std$ or $o \geq m + 2 * std$ (where $o$ is the outlier value, $m$ the group mean, and $std$ the standard deviation).

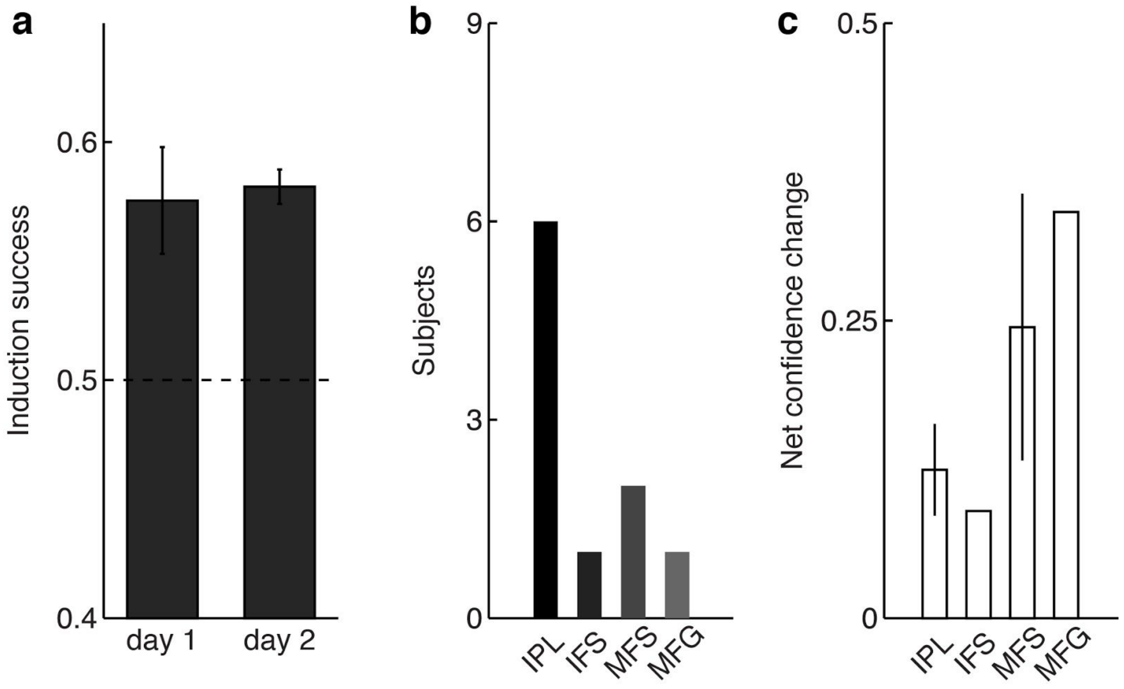


**Supplementary Figure 10. Neurofeedback induction success and ROIs implication in confidence changes**

(a) Induction success (here measured as $p$ for High Confidence DecNef and $1-p$ for Low Confidence DecNef), of the selected best ROI (see Methods section), averaged across both High and Low Confidence DecNef for day 1 and day 2. For each bar, *n* = 10. (b) Number of participants for which IPL, IFS, MFS, MFG, respectively, were selected as the best ROI across High and Low Confidence DecNef. (c) Effect size (net confidence change) associated with each best ROI. Center values correspond to means, and error bars to s.e.m. ROI labels: IPL - inferior parietal lobule, IFS - inferior frontal sulcus, MFS - middle frontal sulcus, MFG - middle frontal gyrus.

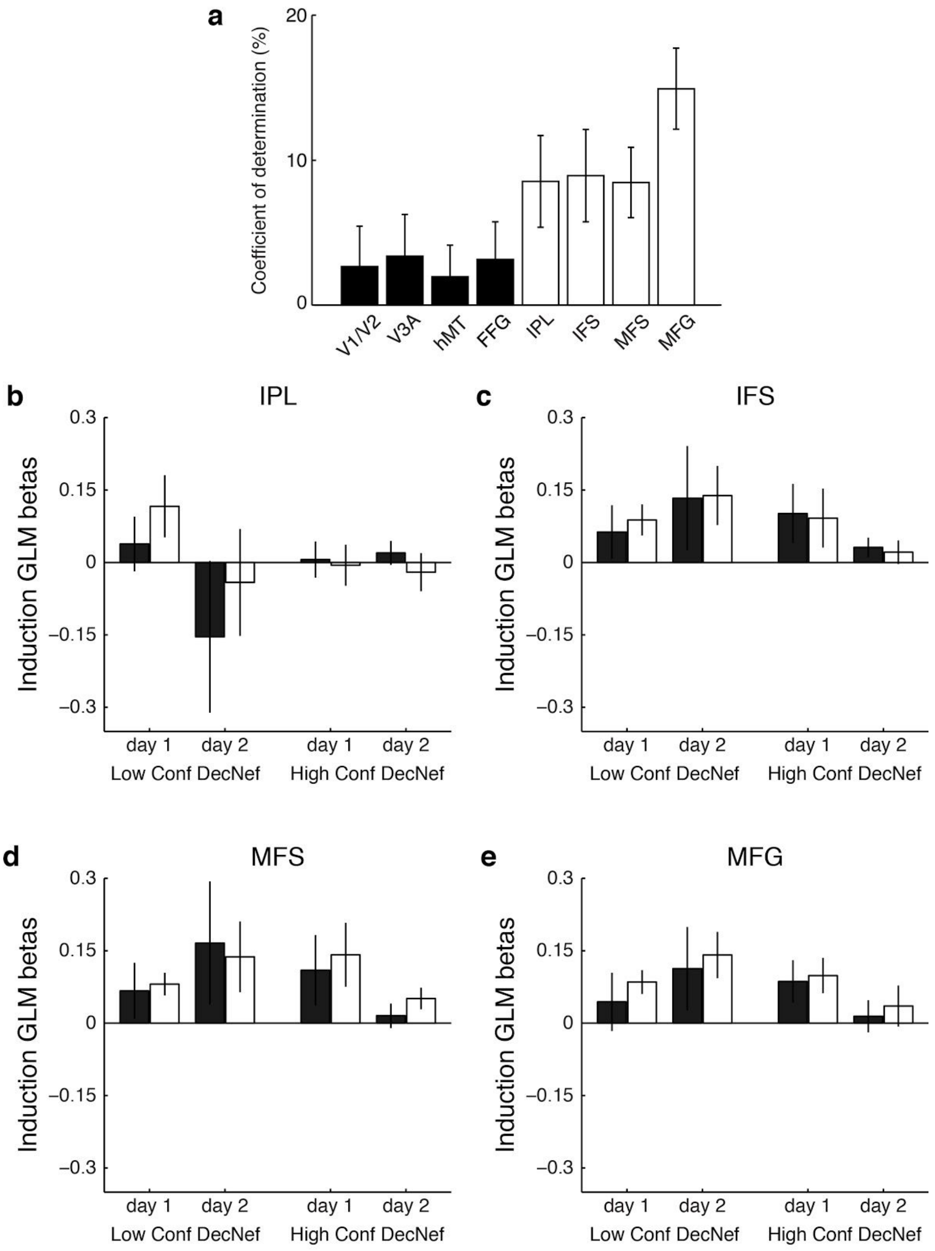


**Supplementary Figure 11. The information communication criterion analysis and univariate contrast between successful and unsuccessful induction trials.**

Information communication criterion analysis for (a) all target ROIs averaged. (b-e) Univariate contrast between successful (black bars) and unsuccessful (white bars) induction trials. During DecNef, induction success was not driven by a general increase or decrease of BOLD signal. Beta estimates from a GLM analysis contrasting successful vs. unsuccessful induction trials are plotted (see Methods section). A general increase or decrease of the BOLD signal would be reflected by the higher positive or negative values of the beta estimates in the GLM analysis for the category in question. Center values correspond to means, and error bars to s.e.m. ROI labels as in Supplementary Figure 10.

# Supplementary Tables

| ROI | Decoding accuracy | ***P*-value (adj. for multiple comparisons)** |
|---|---|---|
| V1/V2 | 57.6%±1.3% | $P<10^{-4}$ |
| V3A | 57.6%±1.5% | $P<10^{-4}$ |
| hMT | 56.2%±1.2% | $P<10^{-3}$ |
| FFG | 58.0%±1.0% | $P<10^{-5}$ |
| IPL | 58.6%±1.0% | $P<10^{-5}$ |
| IFS | 58.4%±1.3% | $P<10^{-4}$ |
| MFS | 55.9%±1.1% | $P<10^{-3}$ |
| MFG | 57.5%±0.9% | $P<10^{-5}$ |

**Supplementary Table 1.** Decoding accuracies (± s.e.m) of correct - incorrect trials classification.

| **ROI** | **Decoding accuracy** | ***P*-value (adj. for multiple comparisons)** |
|---|---|---|
| V1/V2 | 53.1%±2.0% | n.s. |
| V3A | 52.3%±1.8% | n.s. |
| hMT | 55.2%±2.0% | *$P$=0.077 |
| FFG | 52.1%±1.7% | n.s. |
| IPL | 59.3%±2.0% | $P$=0.0016 |
| IFS | 60.7%±1.5% | $P<10^{-4}$ |
| MFS | 58.7%±1.8% | $P$=0.0013 |
| MFG | 62.0%±1.7% | $P<10^{-4}$ |

**Supplementary Table 2.** Decoding accuracies (± s.e.m) of high - low confidence trials classification.

| **ROI** | **Decoding accuracy** | ***P*-value (adj. for multiple comparisons)** |
|---|---|---|
| V1/V2 | 57.9%±1.5% | $P<10^{-3}$ |
| V3A | 54.4%±1.2% | $P=0.0076$ |
| hMT | 53.9%±1.0% | $P=0.0095$ |
| FFG | 55.6%±1.1% | $P<10^{-3}$ |
| IPL | 55.1%±1.0% | $P<10^{-3}$ |
| IFS | 54.2%±1.6% | n.s. |
| MFS | 53.3%±1.3% | n.s. |
| MFG | 54.7%±1.7% | n.s. |

**Supplementary Table 3.** Decoding accuracies (± s.e.m) of left - right perceived motion trials classification.

| ROI | Decoding accuracy | *P*-value (adj. for multiple comparisons) |
|---|---|---|
| V1/V2 | 60.0%±1.0% | $P<10^{-5}$ |
| V3A | 57.3%±0.7% | $P<10^{-5}$ |
| hMT | 57.8%±0.9% | $P<10^{-5}$ |
| FFG | 58.0%±1.1% | $P<10^{-5}$ |
| IPL | 54.8%±0.6% | $P<10^{-5}$ |
| IFS | 58.0%±1.3% | $P<10^{-4}$ |
| MFS | 58.3%±0.9% | $P<10^{-5}$ |
| MFG | 57.5%±0.9% | $P<10^{-5}$ |

**Supplementary Table 4.** Decoding accuracies (± s.e.m) of correct - incorrect trials classification after rectification of signals from left - right perceived motion trials.

| ROI | Decoding accuracy | *P*-value (adj. for multiple comparisons) |
|---|---|---|
| V1/V2 | 51.6%±0.9% | n.s. |
| V3A | 51.7%±1.4% | n.s. |
| hMT | 51.9%±0.8% | n.s. (unc. $P$=0.0393) |
| FFG | 52.8%±1.3% | n.s. (unc. $P$=0.0423) |
| IPL | 51.1%±1.0% | n.s. |
| IFS | 50.5%±1.0% | n.s. |
| MFS | 51.6%±1.3% | n.s. |
| MFG | 52.0%±1.4% | n.s. |

**Supplementary Table 5.** Decoding accuracies (± s.e.m) of high - low confidence trials classification after rectification of signals from left - right perceived motion trials.

| Participant No. | High Confidence DecNef | Low Confidence DecNef |
|---|---|---|
| 1 | Thinking of a scenery or persons with colors, deep inside. | Colored happy thing. Imagined the induction circle is a cookie. |
| 2 | Eating nice food, sports. | Eating nice food, sports. |
| 3 | Thinking about the food to eat after the experiment. Recalling the names of the muscles in the human body. | Mental calculation, puzzle games. |
| 4 | Driving a car, riding a bicycle, playing the piano. | TV game, playing musical instruments. |
| 5 | Calculations, happy memories, delicious food. | Thinking about a kind friend. Thinking about running, moving upper part of the body |
| 6 | Gaze and focus at the screen, look carefully for details. | Gaze and focus at the screen. |
| 7 | Random thoughts, thinking about part-time job. Zoning out. | Random thoughts. Think hard about difficult things. |
| 8 | Concentrate on the induction cue (green circle, gray outline). | Imagining to be looking at many different directions. |
| 9 | Thinking about the graduation thesis. Cooking, planning future activities. | Thinking about classes and tasks at university. Recall the names of other students. |
| 10 | Recalling contents of current research, counting. Trying to think about nothing specific. | Mental calculations, history (historical figures and events). |

**Supplementary Table 6.** Post-experiment interview on induction strategy during High- and Low-Confidence DecNef training sessions.

| **Model (est. parms)** | **Fixed parms** | **AICc** | **$\Delta_i$** | **$w_i$** |
|---|---|---|---|---|
| *Const. Confidence mean* $\left(\overline{X}_1^i\right)$ | k | -70.6447 | 24.3397 | 0 |
| *Const. Confidence [mean* $\left(\overline{X}_2^i, \overline{X}_3^i, \overline{X}_4^i\right)$ *]* | k | -72.1602 | 22.8242 | 0 |
| *Within-week const. confidence* | $k_1$ $k_2$ | -71.7531 | 23.2313 | 0 |
| *Polynomial 1st deg. ($\alpha_1$, $\alpha_2$)* | k | -70.5479 | 24.4365 | 0 |
| *Polynomial 2nd deg. ($\alpha_1$, $\beta_1$, $\alpha_2$, $\beta_2$)* | $k_1$ $k_2$ | -61.8877 | 33.0967 | 0 |
| *Sub-model (Δ)* | $\alpha=1$ $\varepsilon=0$ $\gamma=1$ | -81.6244 | 13.3600 | 0.0005 |
| *Sub-model (Δ, ε)* | $\alpha=1$ $\gamma=1$ | -91.5062 | 3.4782 | 0.0637 |
| *Sub-model (Δ, ε, α)* | $\gamma=1$ | -89.0275 | 5.9569 | 0.0185 |
| *Sub-model (Δ, ε,   )* | $\alpha=0$ | -77.3589 | 17.6255 | 0.0001 |
| *Sub-model (Δ,   , α)* | $\varepsilon=0$ | -91.4285 | 3.5559 | 0.0613 |
| ***Sub-model (Δ, ε,   )*** | $\alpha=1$ | -94.9402 | 0.0442 | 0.3549 |
| ***Sub-model (Δ, ε,   , α)*** | | -93.0529 | 1.9315 | 0.1381 |
| ***Sub-model (Δ, ε, α)*** | $\gamma=0$ | -94.9844 | 0 | 0.3629 |

**Supplementary Table 7.** AICc comparison of various model fits. The most negative AICc value indicates the best fitting model. Because the raw AICc value is not particularly indicative *per se*, we computed both the ΔAICc - the distance between any model and the one with the most negative value, as well as the corresponding Akaike weight $w$. ΔAICc < 2 indicate that the current model has a high likelihood of being the best model under different circumstances, i.e., a new dataset.

# Supplementary Notes

**Supplementary Note 1**
**Nonlinear mathematical modeling**

From a mathematical standpoint, in order to best capture these different components of DecNef effects while accounting for both High and Low Confidence neurofeedback, we fitted a system of nonlinear parametric equations with four global parameters: main effect of change in confidence ($\Delta$), strength of High- relative to Low Confidence induction ($\varepsilon$), maintenance of the newly acquired confidence level over the next session ($\alpha$), and weaker second week effect ($\gamma$). Importantly, we fitted various alternative models, where some of the parameters had fixed values (param = 1 or = 0) to account for full effects, or the lack of effects, in order to compare and infer which aspects of DecNef were likely to play a significant role in determining the resulting confidence changes. Simpler models, that did not assume directionality in confidence changes or other assumptions, included a constant-confidence, a within-week constant confidence, and first-grade polynomial models.

To compare the models we used the corrected Akaike Information Criterion (AICc)[1,2]. Three models could be essentially considered (as good as) the potential best model, since the distance between two of them and the most negative AICc was < 2 (Supplementary Table 6). These were the full model, which estimated all four global parameters, the partial model with $\alpha$=1, and the partial model with $\gamma$ = 0. Importantly, simpler models that did not incorporate changes or directionality such as constant confidence, within-week constant confidence, and first-grade polynomial models, all performed very poorly in fitting the data (Supplementary table 7). These models with $\Delta$AICc > 10 are sufficiently poorer than the best AIC model as to be considered essentially implausible[2]. Most importantly, the three best models possessed non-zero and negative $\varepsilon$, thus the nonlinear modeling results clearly demonstrated that not only High Confidence DecNef but also Low Confidence DecNef induced confidence change in the expected directions. Consequently, the bi-directionality of DecNef was further supported by AICc.

Under model selection uncertainty, with several models having very similar AICc values, a formal solution is to apply model-averaging. Each parameter present in the selected models is estimated according to a weighted average based on the corresponding Akaike weights. For model averaging, we used all models for which ΔAICc < 2, keeping the estimated parameters in the same initial scale and focusing the averaging process on the subset of highly likely models. The delta parameter was 0.37; thus High Confidence DecNef on the first week increased confidence by 0.37 (~20% change in confidence). Alpha was 0.83, hence on average only 17% of the first week effect was lost during the one week interval due to memory decay. Epsilon was -0.35, thus the Low Confidence DecNef effect was opposite in its sign and 35% of the magnitude of that of the High Confidence DecNef. The negative value of the estimated ε again confirmed the bi-directionality of DecNef. Gamma was 0.19, and thus the second week effect was only 19% of that of the first week.

Our nonlinear modeling analyses indicate that there exist both High and Low Confidence DecNef effects, but also anterograde learning interference, and preservation of memory between sessions. Furthermore, the High-Confidence induction effect was larger than the Low-Confidence induction effect. These outcomes explain why the true DecNef effects were partly masked.

# References


1. Akaike, H. A new look at the statistical model identification. *IEEE Trans. Automat. Contr.* **19,** 716–723 (1974).
2. Burnham, K. P. & Anderson, D. R. *Model Selection and Multimodel Inference: A Practical Information-Theoretic Approach*. (Springer Science & Business Media, 2002).
